# PyEvoMotion: a Python tool for population-based time-course analysis of genome evolution

**DOI:** 10.1093/bioinformatics/btag085

**Published:** 2026-02-27

**Authors:** Lucas Goiriz, Guillermo Rodrigo

**Affiliations:** Institute for Integrative Systems Biology (I2SysBio), CSIC—University of Valencia, Paterna, 46980, Spain; Pure and Applied Mathematics University Research Institute (IUMPA), Polytechnic University of Valencia, Valencia, 46022, Spain; Institute for Integrative Systems Biology (I2SysBio), CSIC—University of Valencia, Paterna, 46980, Spain

## Abstract

**Summary:**

We present PyEvoMotion, an open-source Python tool for inferring molecular clock models with time-dependent Gaussian noise from high-throughput genomic datasets. PyEvoMotion features a command-line interface and a modular architecture, allowing seamless integration into larger bioinformatic pipelines. The tool supports customizable filtering, temporal discretization definition, and mutation classification, making it adaptable to diverse research needs. While traditional phylogenetic methods may encounter computational challenges with large datasets, PyEvoMotion can process thousands to millions of sequences to compute statistical parameters associated with a stochastic differential equation model, thereby weighting the genetic variation within the population. Using viral genomic data, we demonstrate its capability to infer evolutionary rates and detect non-Brownian evolutionary motions with subdiffusive behavior. PyEvoMotion shows potential to provide overlooked insights into genome evolution in different contexts.

**Availability and implementation:**

The open source software is available on GitHub at https://github.com/luksgrin/PyEvoMotion and on SourceForge at https://sourceforge.net/projects/pyevomotion.

## 1 Introduction

The study of molecular evolution is a central topic in biology. The molecular clock hypothesis assumes that genes accumulate mutations at a constant rate over time (Kimura 1987). Moreover, under the consideration that most of the accumulated mutations are neutral, the Poisson distribution models the expected variability. The molecular clock hypothesis has become a cornerstone of modern phylogenetic techniques, which are now standard for studying the evolutionary relationships between species and organisms (Kumar 2005).

It has been shown, however, that the simple molecular clock model fails to universally recapitulate evolutionary trajectories. Observations revealed that in some cases mutations do not accumulate at a constant rate (Ayala 1997). This led to the development of relaxed molecular clocks, in which the rates of mutation accumulation are not uniform across lineages (Drummond *et al.* 2006, Lepage *et al.* 2007). Although these clocks have proven to be more accurate in certain cases, they still face difficulties to model, for instance, overdispersed populations (Bedford *et al.* 2008). A proper analysis of the time-dependent distribution of the number of mutations in the population is necessary to understand and eventually predict the evolutionary trajectories that take place in nature.

Although previous studies have attempted to abstract molecular evolution as a type of diffusion process in the sequence space (Kimura 1987, Huynen *et al.* 1996), little attention has been given to the form of the underlying stochastic process. In our previous work, we showed that non-Brownian evolutionary motions occurred within the lineages of a virus, leading to non-Poissonian distributions (Goiriz *et al.* 2023). Here, we present PyEvoMotion, a Python tool aimed to infer a generalized molecular clock model upon bulk genomic data analysis, featuring a command-line interface and enough modularity for integration into larger Python pipelines. PyEvoMotion is intended to complement traditional phylogenetic analyses, focusing on a shorter time scale.

Traditional phylogenetic methods, while powerful, face computational limitations when applied to large datasets. Indeed, analyzing more than $10^{4}$ sequences becomes impractical due to the exponential complexity of reconstructing evolutionary trees (Chor and Tuller 2005). Yet, a recent likelihood-based advance that leverages parsimony-inspired heuristics has enabled phylogenetic inference at much larger scale (De Maio *et al.* 2023). Population-based statistical approaches also provide a viable alternative (Obermeyer *et al.* 2022, Goiriz *et al.* 2023). These methods simplify the representation of evolutionary relationships by focusing on patterns of population genetics rather than exhaustive tree reconstruction based on genetic variation. PyEvoMotion leverages stochastic mathematical modelling to assess evolutionary trends, aiming to process datasets orders of magnitude larger than those typically analyzed. This capability is essential for handling the unprecedented volume of genomic data generated by high-throughput sequencing efforts (Oude Munnink *et al.* 2020).

## 2 Mathematical model

The accumulation of mutations over time in a genome can be modelled in continuous form by the stochastic differential equation


(1) $$\frac{dm(t)}{dt}=\kappa +\xi (t),$$

where m(t) represents the number of mutations at time *t* with respect to a reference sequence, κ is the evolution rate, and ξ(t) is a stochastic process that determines the extent of variability in the population (West and Bickel 1998).

Here, we considered as a null model the case in which ξ(t) is a Gaussian white noise, defined by


(2) 〈ξ(t)〉=0,

(3) $$\langle \xi (t)\xi (t^{\prime })\rangle =D\delta (t-t^{\prime }),$$

where *D* is the stochastic process diffusivity, and δ is the Dirac delta function. Here, 〈·〉 denotes the expectation value (mean) of a random variable, and Δ· the difference between a realization of the random variable and its expected value (i.e. Δm(t)=m(t)−〈m(t)〉). Following some calculations (Wang *et al.* 2022), it can be shown that


(4) 〈m(t)〉=κt

(5) $$\langle \Delta m^{2}(t)\rangle =Dt,$$

where $\langle \Delta \cdot^{2}\rangle$ corresponds to the variance of a random variable. These statistical features characterize a Brownian motion, in agreement with the neutral theory of molecular evolution (Kimura 1987). Clearly, in the case of κ=D, the mean and variance of mutations are equal, leading to a Poissonian regime.

Alternatively, we considered a challenging model in which ξ(t) is a time-dependent Gaussian noise, defined by


(6) 〈ξ(t)〉=0

(7) $$\langle \xi (t)\xi (t^{\prime })\rangle =\frac{1}{2}D\alpha (\alpha -1){\left|t-t^{\prime }\right|}^{\alpha -2}.$$

In this case, α is the diffusion exponent (related to the Hurst exponent) and characterizes the degree of memory in the stochastic process. The process is said subdiffusive when α<1 and superdiffusive when α>1. Then, it can be shown that


(8) 〈m(t)〉=κt

(9) $$\langle \Delta m^{2}(t)\rangle =Dt^{\alpha }.$$

These statistical features characterize a fractal Brownian motion (Fig. S1) (Wang *et al.* 2022). Notably, both the null and challenging models are reconciled when α=1.

## 3 Implementation

### 3.1 Data processing

**Figure 1:**
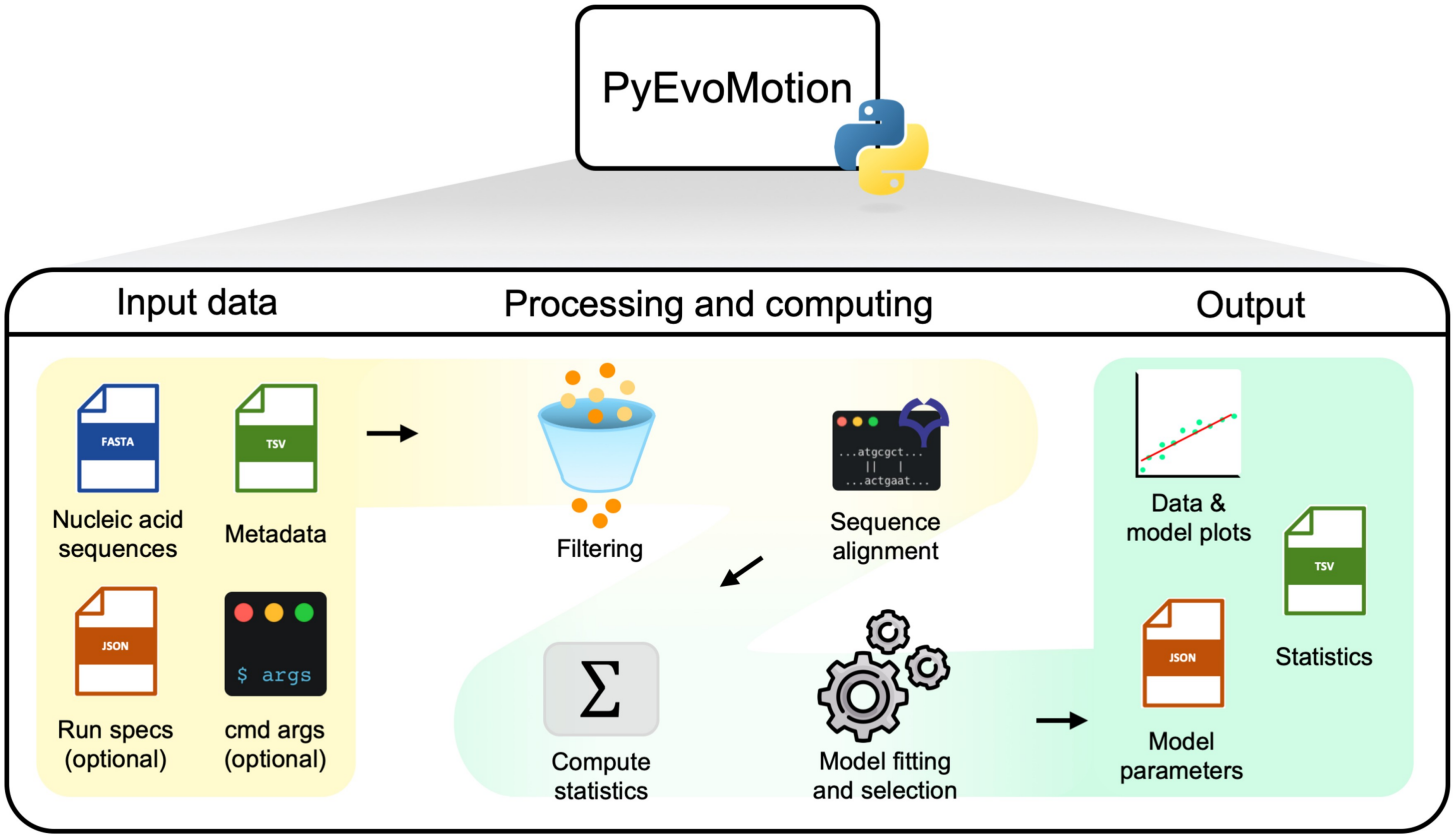
Overview of PyEvoMotion. Mandatory input data include nucleic acid sequences (in.fasta format) and their corresponding metadata (in.tsv format). The metadata must include collection dates, as these are essential for model fitting. Output files include dynamic data representation plots and statistical parameters.

The general workflow of PyEvoMotion is illustrated in Fig. 1. This tool requires two essential input files: a .fasta file containing nucleic acid sequences and a .tsv file with the corresponding metadata. Users can customize their analyses by specifying several parameters and filters.

To begin, the temporal granularity of the analysis can be adjusted by defining the time intervals for grouping sequences and calculating statistics. By default, this interval is set to 7 d. The generation time of the biological entity, or a multiple of it if the time period to be analyzed is long, would be a good option (see Fig. S2 for a comparative analysis). In principle, it would be desirable to have more than 30 sequences per time interval to ensure sufficient statistical power.

Additionally, data filtering options are available to enhance the quality and specificity of the analysis. For instance, the length filter excludes sequences that do not meet a minimum length threshold, thereby removing low-quality genomes (unresolved bases set to a maximum of 1% N). The genome position filter allows users to restrict the analysis to specific genomic regions, which is particularly useful for examining genes or genetic clusters of interest. A date range filter further refines the dataset by limiting the analysis to sequences collected within a specified timeframe.

The tool also enables users to select the types of mutations to include in the analysis. Options include: total (aggregating all mutations without distinction), substitutions, and indels (a combined category of insertions and deletions). These three analyses can be done at once with the option all. Filters based on metadata values provide additional flexibility, enabling users to focus on sequences that meet specific criteria in their non-molecular attributes.

After parsing the sequence data, the reference sequence is extracted, defined as the first entry in the .fasta file. Following the pre-processing step, each sequence is aligned to the reference sequence using the MAFFT algorithm (Katoh and Standley 2013).

Mutation events are identified from the sequence alignments and filtered based on the user-defined mutation types and genomic regions of interest. Statistical analyses are then conducted on the filtered mutation data for each time interval specified, computing mean and variance as


(10) $$\mu_{k}=\frac{1}{N_{k}}\sum_{i=1}^{N_{k}}m_{k,i},$$

(11) $$\sigma_{k}^{2}=\frac{1}{N_{k}-1}\sum_{i=1}^{N_{k}}{\left(m_{k,i}-\mu_{k}\right)}^{2}$$

where $m_{k,i}$ represents the number of mutations observed in the $i^{\text{th}}$ sequence during the $k^{\text{th}}$ time interval, while $N_{k}$ denotes the total number of sequences within that interval. Consequently, $\mu_{k}$ and $\sigma_{k}^{2}$ correspond to the mean and variance of mutations in the $k^{\text{th}}$ time interval, respectively. These statistical measures serve as the basis for fitting a molecular clock model.

Furthermore, PyEvoMotion offers several configurable run-specific parameters to enhance usability and reproducibility. Users can opt to visualize the output data directly, export the plots in PDF format, save the run parameters as a .json file for future reference, or initialize a run using a pre-existing .json file. These features ensure that analyses are both customizable and reproducible, catering to diverse research needs.

### 3.2 Model selection

PyEvoMotion estimates the parameters for both models (i.e. κ and *D* for the null model and κ, *D*, and α for the challenging model), followed by model selection according to the corrected Akaike information criterion (AICc), which includes a small sample size correction to avoid overfitting. For that, the calculated values of mean and variance of mutations at each time are represented, and curves are fitted. A weighted fitting approach is implemented, in which the weight of each time interval reads $w_{k}=\tanh(N_{k}/15)$. That is, data points calculated from few sequences will have a lower contribution (Fig. S3).

κ is directly the slope of the line fitted to the mean of mutations with time (this is the same for both models). The initial mutational load ($m_{0}$) could be non-zero, in which case it would appear in the model as an intercept term. The variance of mutations needs to be rescaled before fitting because the theoretical stochastic processes assume a start from the origin (i.e. they have a defined initial condition), while initial variability may be encountered in real world data. Then, the initial variance is subtracted to all values ($\sigma_{k}^{2}-\sigma_{0}^{2}$) and time is shifted so that $t_{0}=0$. In the case of the null model, *D* is the slope of the intercept-free line fitted to the rescaled variance with time. In the case of the challenging model, a non-linear regression with a power law relationship following the Levenberg-Marquardt algorithm is used to obtain the values of *D* and α.

The confidence interval (CI) for a parameter is computed as the point estimate ± a *t*-multiplier times the standard error, where the *t*-multiplier is taken from a *t*-distribution (degrees of freedom n−2 or n−1 depending on the model). In linear regression, the standard error depends on the noise level in the data (measured by the mean squared error) and on how spread out the predictor values are. In nonlinear models such as a power law, standard errors are obtained from the covariance matrix produced by the nonlinear least-squares fit. This CI calculation is valid under the assumptions that the model is correctly specified, observations are independent, the standard error is well estimated, and the sampling distribution of the parameter estimate is approximately normal.

The fitting of the variance determines the choice of the molecular clock model, which is accomplished by calculating


(12) AICci=n ln(2π)+n ln(RSSin)+n+2pi+2pi(pi+1)n−pi−1,

where RSS${\;}_{i}$ is the weighted residual sum of squares for the $i^{\text{th}}$ model (1 for the null model and 2 for the challenging model), $p_{i}$ is the number of parameters (i.e. $p_{1}=1$ and $p_{2}=2$), and *n* is the number of data points. For models with normally distributed errors, the log-likelihood can be expressed in terms of RSS (Ludden *et al.* 1994).

### 3.3 Modularity

PyEvoMotion includes a command line interface designed for Unix-based systems. Given that most bioinformatic analyses consist of larger workflows, PyEvoMotion provides its outputs in standard formats such as .tsv and .json, which can be easily integrated into existing pipelines. The tool comes also available as a Python module, allowing users to incorporate its functionality and helper utilities into their own Python-based workflows with ease. We also created a Docker image (standalone, executable file) for simplified execution and testing.

Interoperability limitations are minimal but not negligible, as PyEvoMotion relies heavily on MAFFT for sequence alignment. The absence of a proper foreign function interface (FFI) between PyEvoMotion and MAFFT necessitates calling the latter as a subprocess, creating a performance bottleneck. Future versions might mitigate this limitation by introducing a more Python-friendly interface.

The incorporation of alternative mathematical models is possible with little effort in PyEvoMotion due to its modular architecture. Moreover, extended versions of the tool might automatically identify different lineages when analyzing long-time datasets and use piecewise models for the mean and variance of mutations. Importantly, the package implemented continuous integration via GitHub Actions.

## 4 Validation

We generated synthetic sequence data to assess the performance of PyEvoMotion, although without accounting for all layers of uncertainty and complexity of the real world. In particular, we generated evolutionary trajectories considering normal diffusion (i.e. white noise, with κ=0.5 and D=1; dataset S1) and subdiffusion (i.e. time-dependent noise, with κ=0.5, D=1, and α=0.5; dataset S2). We generated 30 different evolutionary trajectories of each type. The tool correctly selected the null model when processing the dataset S1 in 90% of cases. For a representative trajectory, it inferred $\hat{\kappa }=0.500$ [95% CI (0.483, 0.517)] wk${\;}^{-1}$ and $\hat{D}=0.961$ [95% CI (0.864, 1.058)]. Moreover, the tool correctly selected the challenging model when processing the dataset S2 in 100% of cases. It inferred $\hat{\kappa }=0.506$ wk${\;}^{-1}$ [95% CI (0.497, 0.515)], $\hat{D}=1.042$ [95% CI (0.641, 1.443)], and $\hat{\alpha }=0.500$ [95% CI (0.381, 0.619)] for a representative trajectory (Fig. S4). The overall accuracy in model selection was 95% (Fig, S5; see also a report on the variability of the estimations and the empirical coverage of the CIs in Fig. S6). These results help to ensure the reliability of the tool.

The utility of PyEvoMotion was further validated with a real dataset containing whole-genome sequences of the SARS-CoV-2 Alpha variant from the GISAID database (Khare *et al.* 2021). The sequences were divided into two groups based on their country of origin: the United Kingdom (UK) and the United States of America (USA). For each group, we randomly sampled $10^{4}$ sequences (see Fig. S7 for their distribution over time), kept the samples collected between October 2020 and August 2021, and analyzed the number of accumulated mutations over time with respect to the NCBI reference sequence NC_045512.2. All calculations (including data parsing, filtering, sequence alignments, and model fitting) were achieved in about 1 h in a personal computer, showing the potential scalability of the approach.

**Figure 2:**
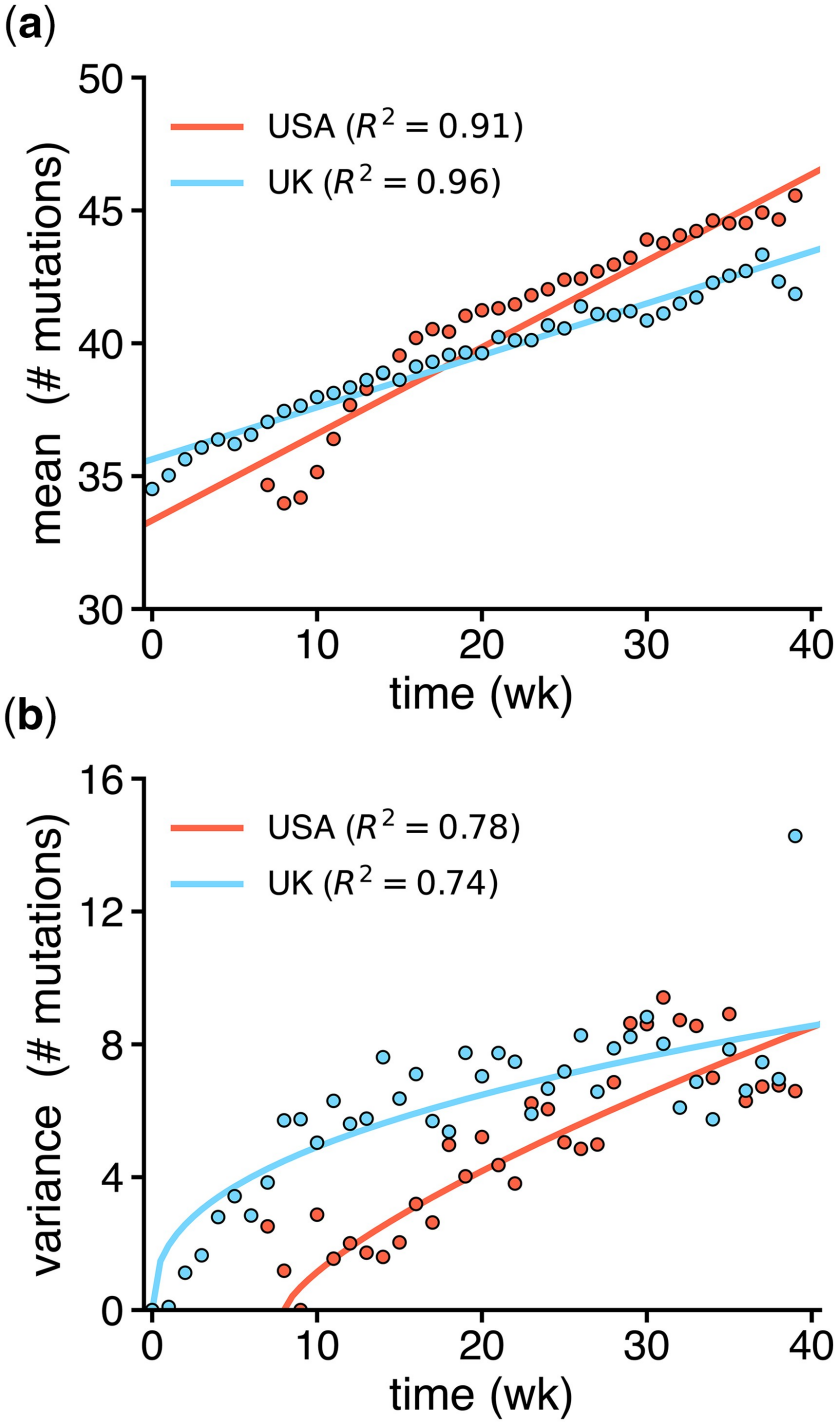
Mutational mean and variance over time in the SARS-CoV-2 Alpha variant genomes from the UK and the USA. a) Mean number of accumulated mutations. b) Scaled variance of the number of accumulated mutations. In the case of the USA, time is not rescaled to the origin for visualization purposes, maintaining the 8 weeks shift with respect to the UK. Points correspond to calculated values from the sequence dataset and lines to inferred molecular clock models.

In the case of the UK, the inferred evolution rate was $\hat{\kappa }=0.195$ wk${\;}^{-1}$ [95% CI (0.183, 0.208)], and the challenging model was the best option, inferring a diffusion coefficient of $\hat{D}=1.950$ [95% CI (1.234, 2.666)] and a diffusion exponent of $\hat{\alpha }=0.401$ [95% CI (0.285, 0.517)]. In the case of the USA, the inferred evolution rate was $\hat{\kappa }=0.326$ wk${\;}^{-1}$ [95% CI (0.288, 0.363)], and the challenging model was also selected, with inferred diffusion parameters of $\hat{D}=0.693$ [95% CI (0.263, 1.122)] and $\hat{\alpha }=0.724$ [95% CI (0.524, 0.924); Fig. 2]. This entails that the evolutionary motion of the Alpha variant was constrained. Moreover, it appears that the virus accumulated mutations on average at a faster rate and in a less constrained way in the USA, arguably due to higher transmission rates resulting from different environmental and social factors (Manathunga *et al.* 2023).

## 5 Conclusions

Here, we present a high-throughput data-processing, open-source, user-friendly software, called PyEvoMotion, to study evolutionary motions under a population-based statistical perspective provided a collection of genomic sequences. PyEvoMotion is designed to be flexible and customizable, offering a wide range of options for data analysis. Such statistical analysis is complementary to phylogenetic tree reconstructions and molecular assays that measure the impact of key mutations (Mlcochova *et al.* 2021).

Nonetheless, our work presents some limitations. In the models, the evolution rate is assumed constant, despite it can vary with time if lineages with higher fitness emerge and even be non-linear if adaptation is the dominant process (Tenaillon *et al.* 2016). This would require applying the date filter to limit the analysis to a subset of sequences, as we did to obtain the results shown in Fig. 2. Moreover, this statistical approach fails to provide meaningful insight if the collection of sequences is not sufficiently large and does not span in time.

In addition to virus evolution, PyEvoMotion might be used to study the tempo and mode of accumulation of mutations in bacteria (Tenaillon *et al.* 2016) or in cancer cells (Borgsmuller *et al.* 2023). Understanding the dynamics of these rapidly evolving biological entities might have biomedical implications.

## Supplementary Material

btag085_Supplementary_Data

## Data Availability

The open source software is available on GitHub at https://github.com/luksgrin/PyEvoMotion and on SourceForge at https://sourceforge.net/projects/pyevomotion. Genomic data used in the validation were extracted from the GISAID database (https://www.gisaid.org) and are available on SourceForge. The package is also published on PyPI (https://pypi.org/project/PyEvoMotion) and Zenodo (https://doi.org/10.5281/zenodo.15477409).
