# Supplementary material for: PyEvoMotion: a Python tool for population-based time-course analysis of genome evolution
Source: Bioinformatics. 2026 Feb 27;42(3):btag085. doi: 10.1093/bioinformatics/btag085 (PMC12960909; doi:10.1093/bioinformatics/btag085)
Supplement: btag085_Supplementary_Data [file btag085_supplementary_data.pdf]

Supplementary data

PyEvoMotion: a Python tool for population-based time-course analysis of genome evolution

Lucas Goiriz, Guillermo Rodrigo

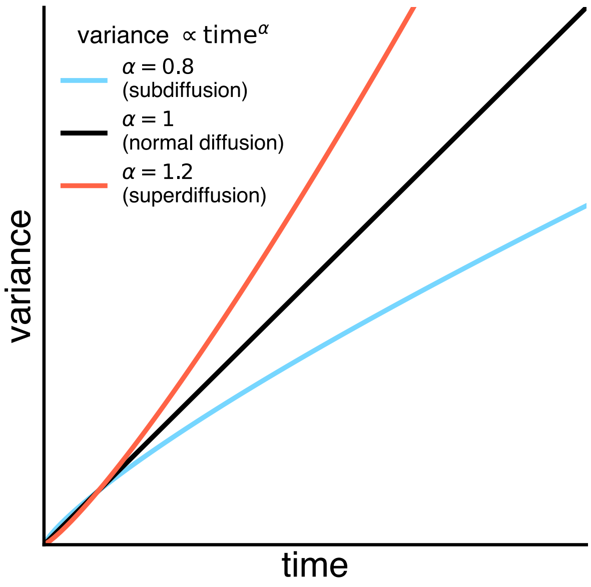

Fig. S1: Illustration of anomalous diffusion behavior (variance vs. time plot).

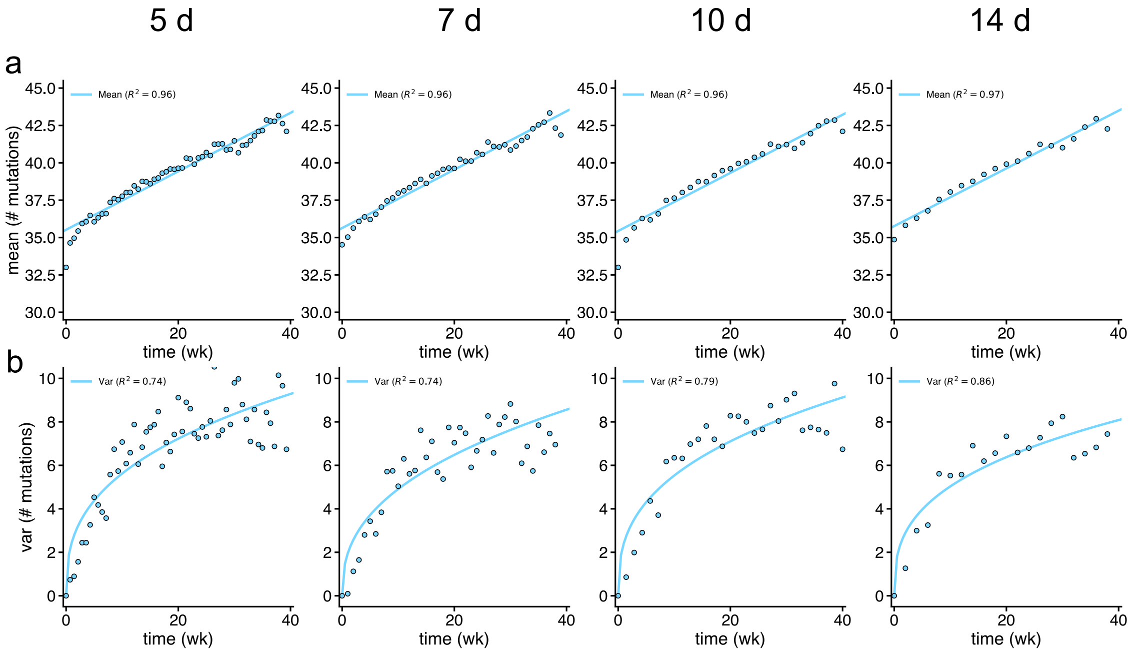

c

	5 d	7 d	10 d	14 d
kappa	0.14	0.20	0.28	0.39
D	2.17	1.95	2.74	2.90
alpha	0.36	0.40	0.36	0.34

Fig. S2: Mutational mean and variance over time in the SARS-CoV-2 Alpha variant genomes from the UK considering different time intervals to group data (5 d, 7 d, 10 d, and 14 d). a) Mean number of accumulated mutations. b) Scaled variance of the number of accumulated mutations. Points correspond to calculated values from the sequence dataset and lines to inferred molecular clock models. c) Parameter values of the inferred models.

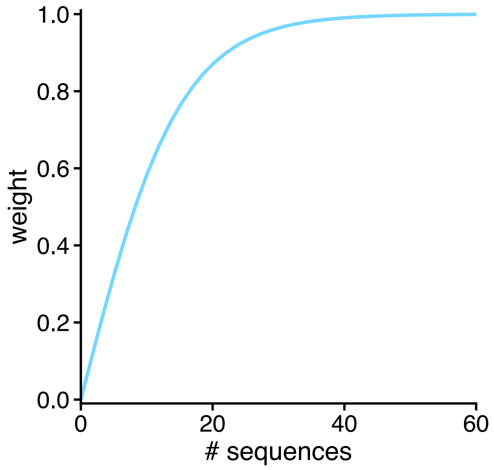

Fig. S3: Weighting function. We selected the functional form $w_k = \tanh(N_k/15)$, with $0 \le w_k \le 1$, to reduce the weight of time intervals with less than 30 sequences, which is a convenient limit to calculate statistical properties with confidence. For example, when $N_k = 15$, $w_k = 0.762$ and when $N_k = 1$, $w_k = 0.067$.

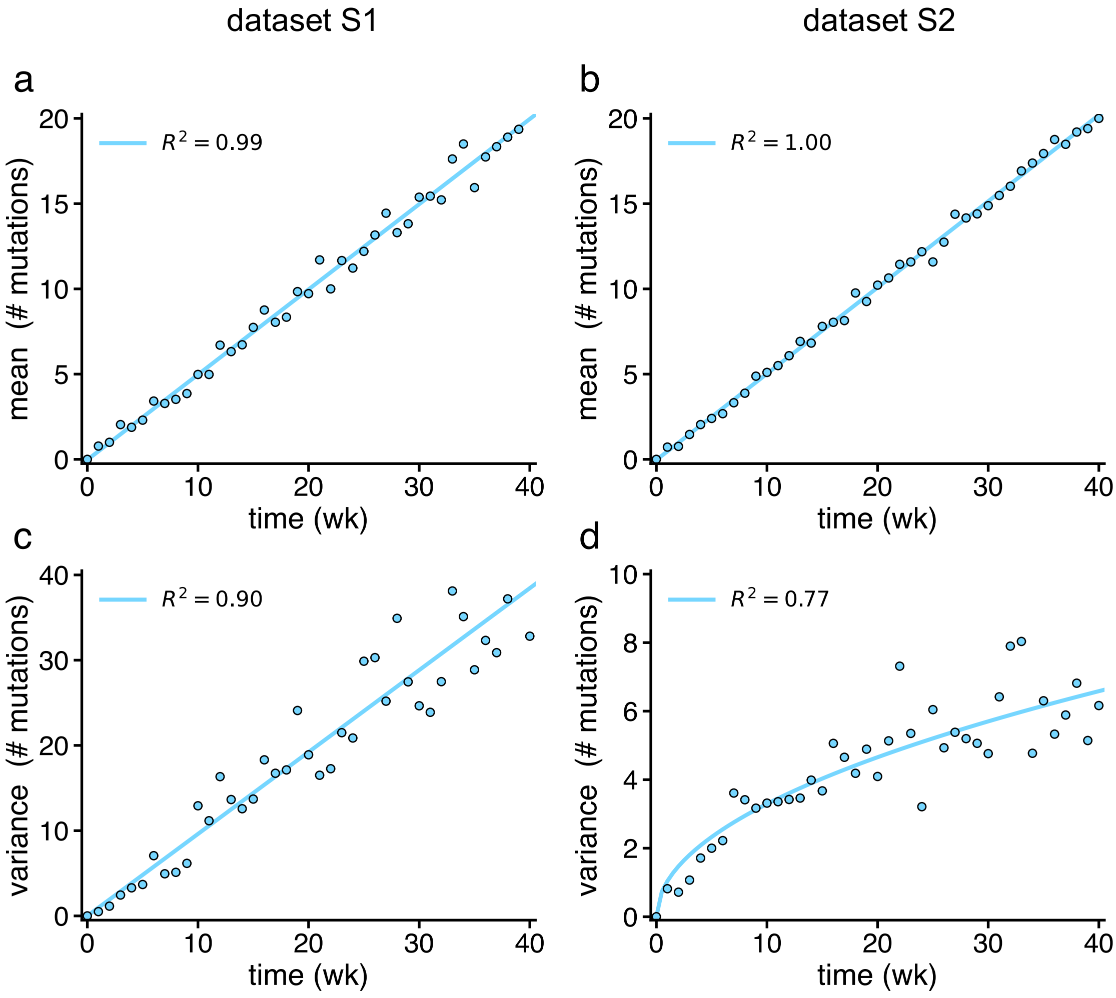

Fig. S4: Mutational mean and variance over time from representative synthetic sequence data (two datasets, one of each type, are considered; S1 for normal diffusion, S2 for subdiffusion). a, b) Mean number of accumulated mutations. c, d) Scaled variance of the number of accumulated mutations. Points correspond to calculated values from the sequence dataset and lines to inferred molecular clock models.

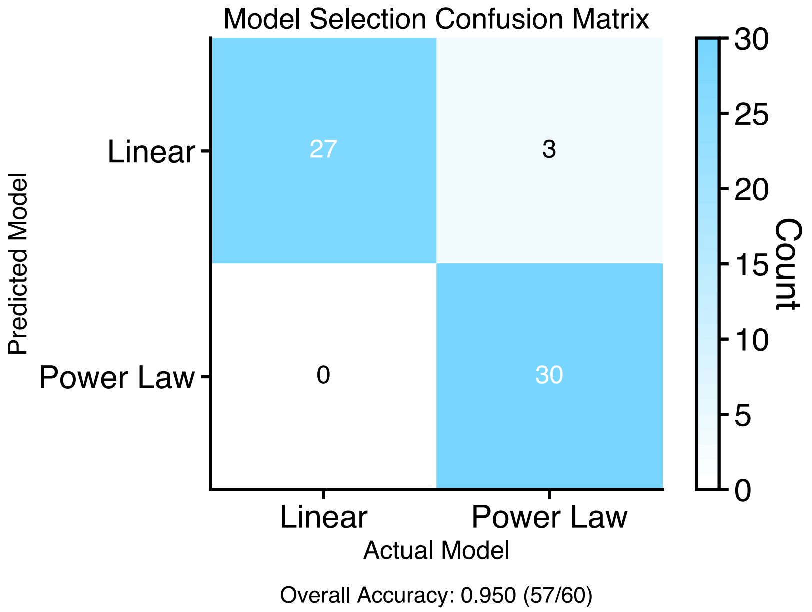

Fig. S5: Confusion matrix relative to model selection with synthetic data.

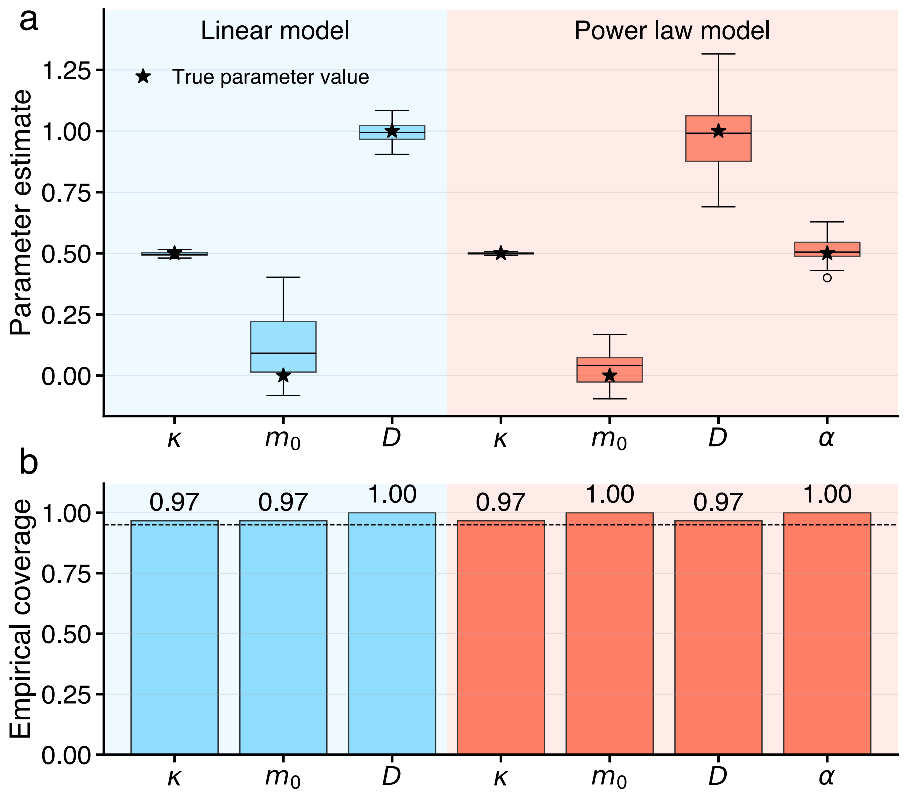

Fig. S6: Computational study of parameter estimation and confidence interval coverage. a) Distributions of parameter estimates across replicates as boxplots generated by considering both normal diffusion (linear model for variance, 30 replicates) and subdiffusion (power-law model for variance, 30 replicates). b) Empirical coverage of the 95% confidence intervals (proportion of replicates in which the interval contains the true value) for each parameter. Dashed line at the nominal 95% level.

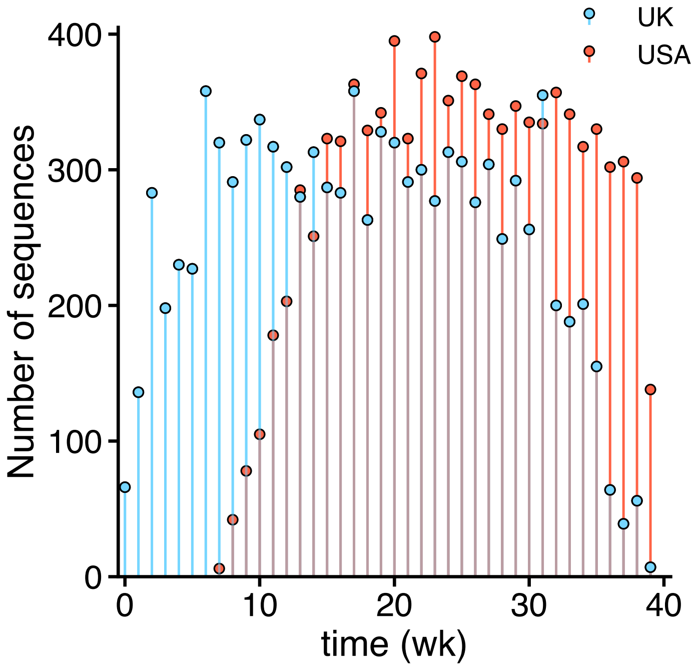

Fig. S7: Number of sequences of SARS-CoV-2 (Alpha variant) per time interval from UK and USA in the sampled datasets of 10^4 elements.
